# Multilevel analysis of discrimination of people living with HIV/AIDS and associated factors in Ghana: demographic health survey of 2022 Ghana data

**DOI:** 10.3389/fpubh.2024.1379487

**Published:** 2024-05-15

**Authors:** Mamaru Melkam, Bezawit Melak Fente

**Affiliations:** ^1^Department of Psychiatry, College of Medicine and Health Science, University of Gondar, Gondar, Ethiopia; ^2^Department of General Midwifery, College of Medicine Health Science, University of Gondar, Gondar, Ethiopia

**Keywords:** Ghana, multilevel, discrimination, stigma, HIV/AIDS, sexually transmitted disease

## Abstract

**Introduction:**

The negative effects of stigma and discrimination in communities and families include medication non-adherence, heightened psychological distress, verbal and physical abuse, a lack of social support, isolation, and dangerous health behaviors such as hiding prescriptions. Despite the huge burden of HIV/AIDS discriminatory attitudes, limited studies were conducted in Ghana. Therefore, this study examines the burden of discriminatory attitudes and their determinant factors on people who are living with HIV/AIDS in Ghana.

**Objective:**

This study aimed to determine the prevalence of discriminatory attitudes and associated factors among people who are living with HIV/AIDS in Ghana based on recent DHS data.

**Method:**

Secondary data analysis was used for this multilevel logistic regression analysis based on the Ghana Demographic Health Survey of 2022. Data extraction, cleaning, and analysis were conducted using Stata version 14. The community of Ghana, from the 15 to 49 age group, was used for this study, with a final sample size of 22,058 participants. Four separate models were fitted, incorporating individual and community levels. Multilevel logistic regression models were calibrated to determine the associated factors at the individual and community level with discriminatory attitudes, with a 95% CI and AOR.

**Results:**

The prevalence of discriminatory attitudes toward people living with HIV/AIDS was 60.92%, with a 95% CI (60.13, 61.70) among Ghana DHS. Lower wealth status, having no comprehensive knowledge of HIV, low educational status at the individual level, and low wealth status at the community level, poorest and poorer [AOR =2.03; 95% CI: (1.04, 3.94)] and [AOR = 2.09; 95% CI: (1.84, 8.65)], respectively, no comprehensive knowledge [AOR = 3.42; 95% CI: (1.74, 6.73)], no and primary education [AOR = 3.18; 95% CI: (2.48, 5.51)] and [AOR = 3.78; 95% CI: (2.68, 5.92)], respectively, at the individual level and low wealth status [AOR = 1.58; 95% CI: (1.00, 2.46)] community level were the associated factors.

**Conclusion:**

The prevalence of discriminatory attitudes toward people living with HIV/AIDS was high (60.92%) in Ghana’s DHS. The associated factors for this study were lower wealth status, having no comprehensive knowledge of HIV, and low educational status at the individual level.

## Introduction

The human immunodeficiency virus (HIV) is a serious global public health concern that is particularly prevalent in Sub-Saharan Africa (1, 2). The majority of PLHIV commonly experience discrimination and stigma. There are various types of stigma: “self-stigma,” which refers to a negative perception of oneself experienced by people living with HIV (PLHIV); “anticipated stigma,” which is the belief held by PLHIV that they will face discrimination or be negatively judged upon disclosing their status; and “external or enacted stigma,” which is an actual instance of discrimination that PLHIV has to deal with (3, 4). Other members of the community’s discriminatory behaviors included moving away from the chairs next to PLHIV, refusing to sit close to them, and acting as though they were afraid to make direct physical contact with them (4). Prejudice, stereotyping, and discrimination against PLHIV are the three main ways that stigma mechanisms demonstrate how uninfected people respond when infected persons come to pass. Prejudice is the unfavorable feelings that non-infected individuals have toward and about PLHIV, including feelings of disgust, rage, and fear (4, 5).

Stigma can result in discrimination along with other civil rights breaches that have a profoundly negative impact on the welfare of people who live with HIV (6). Discrimination is defined as a distinct act or conduct against the stigmatized individual based on those beliefs and perceptions that hinder the achievement of universal access to HIV/AIDS prevention, treatment, care, and support services (7). HIV-related prejudice and stigma are making it more difficult for people to obtain HIV-related services and support programs (8). Beyond the illness itself, there has been widespread prejudice, severe suffering, and abuses of human rights (9). Numerous scholars and advocates have contended that the stigma surrounding HIV/AIDS is an enduring issue that diminishes the efficacy of preventative measures, deters individuals at risk of infection from testing for HIV, and creates obstacles to HIV-related care and assistance (10, 11). Chronic stress discrimination and stigma may make people more vulnerable to poor physical and mental health.

According to the most recent UNAIDS data, 79.3 million people worldwide have contracted the virus since the outbreak started, of whom 36.6 million have passed away (12). The yearly incidence of new HIV infections in girls and women has decreased rapidly globally, accounting for 27% of cases, according to the UNAIDS report (13). According to the most recent assessment in 2020, 240,000 new cases of AIDS and 130,000 AIDS-related deaths (3). In second position with 14.3% of the population living with the disease, are who account for 16.2% of all AIDS cases in the country (14). Furthermore, Africa had the largest burden of HIV, with over 25 million people afflicted (15). HIV/AIDS is a major problem in African nations, especially in the East and South, where it accounts for two-thirds of the total number of infected people (16). The prevalence of HIV discrimination in low- and middle-income countries was 47.08% in sub-Saharan Africa, 6.3 to 29.9% in Ghana, and 62.66% in Ethiopia, according to Demographic and Health Survey data (17, 18).

Discrimination from the community is a further contributor that lowers the level of living for people living with HIV/AIDS. These individuals, together with their relatives and healthcare providers who support them, may lose their jobs or income, become estranged from their surroundings, or become unable to participate in society as contributing members (19). There are a lot of factors that were associated with discrimination against PLHIV; participants’ advanced age, marital status, a greater level of poverty, awareness of HIV, unsafe sexual activities, and medical insurance coverage were associated factors with HIV discrimination in low- and middle-income countries (20–22). In low- and model-income countries, factors that were associated with the discriminatory attitude of people who live with HIV included comprehensive knowledge of HIV, educational level, mass media exposure, economic status, and ever being tested (17–19).

Developing and planning effective policies, programs, and strategies targeted to reduce the discrimination burden on clients who have suffered from HIV/AIDS are mandatory. The presence of discriminatory attitudes might cause a burden on clients with mental and physical health. Additionally, clients who have lived with HIV/AIDS could not be checked themselves due to the fear of this discriminatory attitude. Even though many people living with HIV/AIDS have numerous hazards that result in stigma and discrimination, studies that show the individual- and community-level burden in the national dataset are limited. Therefore, this study aimed to reveal the prevalence and determinants of factors associated with discriminatory attitudes of people who are living with HIV/AIDS in Ghana from the recent Demographic Health Survey (DHS) of 2022.

## Methods

### Study design and data source

A secondary data analysis in Ghana from the recent Demographic Health Survey (DHS) of 2022 was used. The recent Ghana 2022 Demographic Health Survey data were used for this study. A total of 618 clusters were selected from the Ghana Household Health Survey framework using the equal probability selection method. The Ghana DHS provided datasets on men, women, children, births, and households for the survey. The Individual Record dataset (IR file) was the data extracted for this survey. Participants, who were sexually active between the ages of 15 and 49, from the Ghana community, serve as the source populations. The final weighted sample size of this secondary data analysis was 22,058 participants, including men and women from the Ghana DHS 618 clusters or EAs. Detailed information on the data is available on the official link^1^ (23).

### Variables of the study

#### Outcome variables

Our outcome variable is the discriminatory attitude of people who live with HIV; it was a dichotomic variable from the Ghana DHS data. Discriminatory attitude was measured by the following query: study participants who responded no for either of the following questions were considered to have discriminatory attitudes (23).

1. Children with HIV should be allowed to attend school with children without HIV.
2. Would you buy vegetables from a vendor with HIV?

### Independent variables

The assessment of the discriminatory attitude of people who are living with HIV/AIDS included both individual and community-level variables. The independent variables were extracted from the Ghana DHS 2022 data and incorporated; age, occupation, sex of the respondent, religion, ethnicity, marital status, comprehensive knowledge of HIV, educational level, residence, mass media exposure, economic status, ever being tested of HIV, and unsafe sexual behavior. Comprehensive knowledge of HIV/AIDS was measured by the following six yes/no questions and we calculated it to decide, based on the previous study cutoff point: (1) We can get HIV by witchcraft or supernatural means. (2) Consistent use of condoms during sexual intercourse can reduce the chance of getting HIV. (3) Having just one uninfected faithful partner can reduce the chance of getting HIV. (4) Can get HIV from mosquito bites. (5) Can get HIV by sharing food with a person who has HIV/AIDS. (6) A healthy-looking person can have HIV. If the respondents answered all six questions properly, they were considered to have comprehensive knowledge. The determinants of discriminatory attitudes of people with HIV/AIDS at the community-level variable extraction in this study are incorporated: community residency (urban and rural), community educational level (low and high), community wealth status (low and high), and community media exposure (low and high). The community-level variables are calculated based on their cluster by running them together in Stata, and the proportion of the community-level variable is calculated in an Excel spreadsheet. Finally, it is categorized based on its normality. We used the mean for normal distributed variables and the median for skewed distributed variables to calculate community-level variables.

### Data management and analysis

Data extraction, coding, cleaning, and analysis were conducted by using Stata version 14 software. The descriptive statistics of the variables were conducted and reported based on frequency and percentage in a table and text. The non-proportionate allocation of the analysis and the sample’s representativeness were performed using sample weight with cluster. To maintain the hierarchical structure of the gathered data, a mixed multilevel analysis was performed.

Multilevel bivariable logistic regression analysis was conducted to determine the associated variables to be entered into multivariable analysis with a *p*-value of less than 0.25. Multilevel multivariable logistic regression analysis was used to determine the statistically significantly associated variables with a *p*-value of less than 0.05, and an adjusted odds ratio (AOR) with a 95% confidence interval (CI) was calculated. For the multivariable multilevel logistic regression analysis, four model analyses were conducted. The initial model, also known as the null model, was run without the use of any explanatory variables. Only the individual-level variables were fitted in the second model; in the third model, only community-level variables were included; and both individual and community-level variables were fitted in the fourth model.

The outcome variable measures of variation or random effects were estimated by the median odds ratio (MOR), intra-class correlation coefficient (ICC), and proportional change in variance (PCV). Deviance and the Akaike information criterion (AIC) were conducted to compare and assess the fitness of the models; the model with the lowest score was deemed to be the best fit. PCV determines the variation in the prevalence of discriminatory attitudes explained by the fitted model. PCV= $\frac{\mathrm{Vnull}-VA}{\mathrm{Vnull}}*100\%\text{,}$ (Vnull is the variance of the null model, and VA is the variance of the cluster). Additionally, the intra-class correlation coefficient (ICC) was used to measure the degree of heterogeneity of discriminatory attitude between the clusters (the proportion of the overall observed individual variance in discriminatory attitude that can be attributed to differences between clusters) calculated formula ICC= $\frac{VA}{VA+3.29}*100\%$ . The median odds ratio (MOR) was used to quantify the variation of discriminatory variables across clusters: MOR = e^0.95√VA^ (24). Finally, the AOR with 95% CI was calculated, and variables statistically significantly associated with discriminatory attitudes were determined with a *p*-value less than 0.05.

## Results

### Socio-demographic characteristics of respondents

A total of 22,058 study participants were included in this study. Of the study participants, 15,014 (53.08%) were females and 12,112 (54.90%) were in secondary education. Of the study participants, 7,339 (33.27%) were Pentecostal religion followers and 7,629 (34.58%) were from Akan ethnicity. Of the study participants, 17,852 (80.90%) had occupations and 10,563 (47.88%) were married. Of the total study participants, 18,177 (82.40%) had no comprehensive knowledge about HIV/AIDS. Of the study participants, 11,445 (51.88%) were from rural areas. Of the total study participants (18,566), 84.17% were not tested for HIV in their lives (Table 1).

**Table 1:** Socio-demographic characteristics of study participants of Ghana DHS (*n* = 22,058).

Variables	Category	Weighted frequency (*n* = 22,058)	Percentage (%)
Age	15–19	4,265	19.33
	20–24	3,692	16.73
	25–29	3,268	14.81
	30–34	3,127	14.17
	35–39	2,827	12.81
	40–44	2,357	10.68
	45–59	2,522	11.43
Sex	Female	15,014	53.08
	Male	7,044	46.92
Educational status	No education	4,543	20.59
	Primary	3,168	14.36
	Secondary	12,112	54.90
	Higher	2.235	10.13
Religion	Catholic	2,440	11.06
	Pentecostal	7,339	33.27
	Christian	2,969	13.46
	Islam	5,987	27.14
	Others religion*	3,323	15.06
Ethnicity	Akan	7,629	34.58
	Ewe	2,406	10.90
	Mole-Dagbani	5,835	26.45
	Gurma	2,151	9.75
	Others ethnicity**	4,037	18.30
Work status	Have job	17,852	80.90
	No job	4,206	19.10
Marital status	Never in union	7,963	36.10
	Married	10,563	47.88
	Widowed/divorced	3,532	16.02
Wealth status	Poorest	5,515	25.00
	Poorer	4,919	22.30
	Middle	4,326	19.61
	Richer	3,921	17.77
	Richest	3,377	15.30
Comprehensive knowledge	Yes	3,881	17.60
	No	18,177	82.40
Ever being tested	Yes	3,492	15.83
	No	18,566	84.17
Community level Wealth index	High	313	50.65
	Low	305	49.35
Community level Media exposure	High	306	49.51
	Low	312	50.49
Community level Educations	High	476	77.02
	Low	142	22.98
Residency	Urban	10,613	48.12
	Rural	11,445	51.88

Other religion*, Anaglican, Methodist, Presbyterian, Traditionalist, and No religion.

Other ethnicity**, Mande, Gursi, Guan, and Ga/Dangme.

### Prevalence of discriminatory attitude toward people living with HIV

The prevalence of discriminatory attitudes toward people living with HIV/AIDS was 60.92% with a 95% CI (60.13, 61.70) in the Ghana Demographic Health Survey of 2022.

### Random effect model and model fitness

The clustering effect of the data is assessed because the DHS data are hierarchical. The ICC of the null model (model 1) was 21.20% variations of the discriminatory attitude among the cluster and the remaining 78.8% were due to variations of individuals. The null model’s MOR of discriminatory attitude was 2.45%, demonstrating that there was diversity among the clusters. From each of the two clusters, if we selected a single participant randomly, the odds of having a discriminatory attitude in that person were 1.6 times higher from the higher risk cluster than the cluster with a lower risk. The fitness of the model was determined by the deviance and AIC value; therefore, model IV was the best-fitted model for this study (Table 2).

**Table 2:** Multilevel multivariable logistic regression analysis of Ghana demographic and health survey data analysis (*n* = 22,058).

Variable	Category	Model I	Model II	Model III	Model IV
Age	15–19		1.55 (1.18, 2.04)		2.05 (0.72, 5.80)
	20–24		1.37 (1.07, 1.74)		1.40 (0.57, 3.44)
	25–29		1.62 (1.28, 2.04)		1.69 (0.73, 3.87)
	30–34		1.16 (0.93, 1.46)		0.80 (0.37, 1.73)
	35–39		0.99 (0.79, 1.24)		0.95 (0.43, 2.08)
	40–44		0.91 (0.72, 1.14)		0.93 (0.38, 2.26)
	45–49		1		1
Sex of household	Male		1		0.94 (0.61, 1.44)
	Female		0.95 (0.85, 1.07)		1
Wealth status	Poorest		2.29 (1.78, 2.94)		**2.03 (1.04, 3.94)**
	Poorer		1.85 (1.52, 2.24)		**2.09 (1.84, 8.65)**
	Medium		1.52 (1.29, 1.81)		1.83 (0.83. 4.06)
	Richer		1.09 (0.93, 1.28)		1.24 (0.77, 2.00)
	Richest		1		1
Comprehensive knowledge of HIV	Yes		1.40 (1.21, 1.61)		**3.42 (1.74, 6.73)**
	No		1		1
Mass media exposure	No		1.45 (1.16, 1.79)		0.51 (0.99, 2.65)
	Yes		1		1
HIV tested	Yes		1		1
	No		0.91 (0.80, 1.02)		1.13 (0.74, 1.73)
Education status	No education		8.21 (6.09, 11.07)		**3.18 (2.48, 5.51)**
	Primary		9.09 (6.91, 11.96)		**3.78 (2.68, 5.92)**
	Secondary		4.46 (3.55, 5.60)		1.05 (0.67, 1.43)
	Higher		1		1
Marital status	Not in union		1		1
	Married		1.17 (0.97, 1.41)		1.05 (0.53, 2.07)
	Widowed/divorced		1.32 (1.12, 1.56)		1.48 (0.79, 2.77)
Work status	Not working		1.10 (0.95, 1.28)		0.91 (0.51, 1.63)
	Working		1		1
Community-level variables					
Residence	Urban			1	1
	Rural			1.76 (0.94, 3.29)	1.25 (0.72, 2.18)
Media exposure	High			1	1
	Low			0.31 (0.75, 2.05)	1.01 (0.65, 1.57)
Educations	High			1	1
	Low			1.20 (0.74, 1.95)	0.74 (0.44, 1.25)
Wealth status	High			1	1
	Low			1.63 (1.06, 2.49)	**1.58 (1.00, 2.46)**
	Likelihood ratio	−9308.1302	−4355.2144	−387.69684	−348.21964
	ICC	0.2120946	0.0575693	0.1252664	0.060895
	Deviance	18616.26	8710.4288	775.39368	707.9833
	AIC	2215.789	2086.526	17955.27	17952.27
	PCV	Ref	32.70%	41.82%	47.35%
	MOR	2.45			

ICC, Intra-class correlation; MOR, Median odds ratio; AIC, Akaike Information Criterion; PCV: Proportional change in variance. Bold indicates significantly associated variables.

### Factors associated with discriminatory attitude

In multilevel bivariable logistic regression analysis, age, educational level, sex of the respondent, wealth status, marital status, comprehensive knowledge of HIV, ever being tested for HIV, occupation, and mass media exposure were associated factors with discriminatory attitude toward people living with HIV/AIDS at the individual level. Wealth status and residency were variables that were associated with discriminatory attitudes toward people living with HIV/AIDS at the community level.

In multilevel multivariable logistic regression analysis at the individual level, poor and poorest wealth status, no comprehensive knowledge of HIV, low educational status, and low wealth status at the community level were associated factors with people living with HIV/AIDS. The odds of the development of discriminatory attitude toward people living with HIV/AIDS were 2.03 and 2.09 times higher among the poorest and poorer as compared to the richest participants [AOR =2.03; 95% CI: (1.04, 3.94)] and [AOR = 2.09; 95% CI: (1.84, 8.65)], respectively. The existence of discriminatory attitude was 3.42 times higher among participants who have no comprehensive knowledge of HIV as compared to others who have comprehensive knowledge [AOR = 3.42; 95% CI: (1.74, 6.73)]. Discriminatory attitude was 3.18 and 3.78 among participants who have not educated and primary education status as compared to higher educational status [AOR = 3.18; 95% CI: (2.48, 5.51)] and [AOR = 3.78; 95% CI: (2.68, 5.92)], respectively. The community-level occurrence of discriminatory attitude was 1.58 times higher among low wealth status than high status [AOR = 1.58; 95% CI: (1.00, 2.46)] (Table 2).

## Discussion

The objective of this study was to determine the discriminatory attitude of people living with HIV/AIDS in Ghana DHS 2022 data. The prevalence of discriminatory attitudes toward people living with HIV/AIDS was 60.92% with a 95% CI (60.13, 61.70), according to Ghana DHS 2022 data. This finding is lower than other studies conducted in Ethiopian DHS (62.66%) (18). This discrepancy in attitude might be due to the differences in socioeconomic status, level of education, and cultural myths among countries (18). In other words, this finding is greater than studies conducted in four African countries (34.6%) (11) and China (42%) (25). This discrepancy might be due to the fact that residents of these impoverished areas have limited access to healthcare, media, and educational resources. The majority of pastoralist communities reside in areas where it has been extremely challenging to provide health and other developmental services, thereby preventing them from knowing about HIV (25).

Related to factors associated with the discriminatory attitude of people living with HIV/AIDS was the low wealth status at both the individual and the community level. This finding coincided with other studies conducted in Ethiopia (17), Tajikistan (26), and Pakistan (27). The possible reason for these associations might be the effect of high socioeconomic backgrounds being more likely to be well-educated, have better access to the media, possess more current information, and be more aware of and cautious about their health issues (17). The other reason for this association could be the opposite effect of the values and norms of society being over-dominated by the wealthiest person rather than the low socio-economic status individual (19). The other factors that were associated with the discriminatory attitudes of people living with HIV/AIDS were being not educated and having only primary education. This finding was consistent with other studies conducted in Ethiopia (28, 29), Pakistan (30), and Ghana (31). The probable reason for this association could be that people with higher levels of education are assumed to be more aware of HIV and approach it with helpful intentions. Additionally, they have access to a larger platform for learning about HIV testing, stigma, and activities that know the transmitted method (31). Another possible reason for the associations was that, according to a study in Pakistan, participants with lower education levels are less likely to have positive opinions toward people living with HIV, possibly due to their critical thinking (30). Furthermore, it might be the case because education is a potent instrument that modifies people’s views by fostering a deeper comprehension of HIV/AIDS. Moreover, educated people are more accepting of those who are HIV/AIDS positive and demonstrate a willingness to respect the survivors’ right to survival and to engage with others (29). Positive sociocultural change can be facilitated by education, which also assists people in dispelling myths and conventional wisdom about the pandemic and those who are HIV/AIDS positive (32). The other reason for the association of this factor could be the effect of education on having good knowledge about the virus, which changes the myths and misconceptions about people living with HIV/AIDS (30). Successful attitude change in educational initiatives to raise awareness of the condition and modify people’s attitudes and behaviors toward HIV/AIDS and employing tried-and-true behavior modification techniques, such as creating exemplars for others to follow from the educated.

Comprehensive knowledge about HIV/AIDS was another factor associated with discriminatory attitudes toward people living with HIV/AIDS. This association was associated with other studies conducted in Ethiopia (17, 29), Indonesia (3), and Ghana (31) The reason for the associations could be the effect of the most obvious explanation, which is that stigmatizing behaviors and discriminatory attitudes toward HIV-positive individuals are reduced when others are informed accurately about HIV transmission techniques and the misconceptions surrounding AIDS transmission (17). Enhancing a thorough understanding of HIV could be crucial for lowering stigma and increasing test-take rates. Knowing about the nature, transmitted methods, and prognosis of HIV/AIDS is very important to having a good attitude and not discriminating toward people living with the virus (3). This suggests that HIV knowledge should include explaining HIV transmission, mitigation, and care in addition to spreading awareness of the virus (31).

## Conclusion and recommendations

The prevalence of discriminatory attitudes toward people living with HIV/AIDS was high (60.92%), according to Ghana DHS 2022 data. The associated factors for this study were lower wealth status, having no comprehensive knowledge of HIV, low educational status at the individual level, and low wealth status at the community level. To reduce the discriminatory attitude toward people living with HIV/AIDS, it is recommended to increase comprehensive knowledge of HIV, promote education, and increase the socioeconomic level. Ghanaian clinicians are encouraged to raise awareness and promote non-discrimination against people with HIV/AIDS. The government is recommended to give hard and fast rules and regulations against discrimination.

## Data availability statement

The datasets presented in this study can be found in online repositories. The names of the repository/repositories and accession number(s) can be found in the article/supplementary material.

## Ethics statement

The studies involving humans were approved by IRB-approved procedures for DHS public-use datasets do. The studies were conducted in accordance with the local legislation and institutional requirements. The participants provided their written informed consent to participate in this study. Written informed consent was obtained from the individual(s), and minor(s)' legal guardian/next of kin, for the publication of any potentially identifiable images or data included in this article.

## Author contributions

MM: Investigation, Methodology, Writing – original draft, Writing – review & editing. BF: Formal analysis, Writing – original draft, Writing – review & editing.

## Footnotes

^1^ http://www.dhsprogram.com/
